# Supplementary material for: Striatal M4 Receptors Mediate M4 PAM Antipsychotic-like Efficacy Independently of Normalizing Striatal Dopamine Release
Source: bioRxiv. 2026 Oct 27:2026.10.26.761976. Preprint. [Version 1] doi: 10.64898/2026.10.26.761976 (PMC13733948; doi:10.64898/2026.10.26.761976)
Supplement: Supplementary file 1 [file NIHPP2026.10.26.761976V1-supplement-1.pdf]

bioRxiv preprint doi: https://doi.org/10.64898/2026.10.26.761976; this version posted October 27, 2026. The copyright holder for this preprint (which was not certified by peer review) is the author/funder, who has granted bioRxiv a license to display the preprint in perpetuity. It is made available under a CC-BY 4.0 International license.

Supplemental Methods

Stereotaxic Surgery

Mice were anesthetized with an isoflurane-oxygen mixture (1.5-2.5% isoflurane) and mounted in stereotaxic apparatus (Stoelting). Viral vectors were injected using a Hamilton syringe. To secure cannulas, two screws, driven into pre-drilled holes, and a base layer of dental cement were applied to the skull for adhesion (Metabond powder, 1861068, Metabond base, 1864477, Amalgambond PLUS Catalyst, 1861119, Henry Schein), and the cannula was secured in place with Integrity dental crown material (Henry Schein 2226781). All mice received post-operative subcutaneous injections of carprofen at 50 mg/kg, with additional doses at 24 and 48 hours post-surgery. Mice were allowed 3+ weeks for healing and expression of virus before habituation and experimentation. At the end of experiments animals were sacrificed and brains were removed for immunohistochemistry analysis to confirm viral expression, cannula placement, and in some cases M4 muscarinic receptor deletion as outlined below. Any mice with insufficient viral transduction or cannula placement outside of the DS were excluded from analysis.

Habituation

Prior to all experiments, mice underwent 3 consecutive days of habituation. Each day, mice were handled and weighed, connected to the fiber-photometry patch cable, administered a saline injection, and placed in the open-field arena for 10 min.

Fiber Photometry

The setup utilized low-autofluorescence FC/PC and FC/PC-to-ferrule patch cables (Thorlabs) along with mirrorboxes and pig-tailed rotary joints (Doric).

Assessment of Basal Striatal Dopamine Release and Locomotion:

Dose-response experiments utilized a 10-min baseline prior to treatment injection, followed by 45 min of recording post-injection. Dosages given were 0, 0.3, 1, and 3 mg/kg. Tonic DA with DETQ experiments utilized a 30-min baseline for maximal photobleach stabilization for optimum normalization when calculating Z-score.

bioRxiv preprint doi: https://doi.org/10.64898/2026.10.26.761976; this version posted October 27, 2026. The copyright holder for this preprint (which was not certified by peer review) is the author/funder, who has granted bioRxiv a license to display the preprint in perpetuity. It is made available under a CC-BY 4.0 International license.

Locomotion analysis: The DeepLabCut model was trained at 150,000 iterations. Pose-estimation data were filtered. Distance traveled was calculated from the centroid position, then analyzed using DeepLabCut-Analysis-Jupyter-Scripts, publicly available code developed by Farhan Augustine (10.5281/zenodo.10866995). Time in the center was defined as the time during which both the headbase and centroid were located within the central arena zone, defined as a circle centered in the arena with a radius equal to one-half of the arena radius.

Tissue Processing

Immunohistochemistry: After perfusion with 4% PFA, brains were removed, post-fixed overnight in 4% PFA, and cryoprotected by sequential overnight incubation in 15% and 30% sucrose. Brains were sectioned on a freezing microtome. Sections were permeabilized in permeabilization buffer (0.5% Triton X100 in PBS) for 30 min prior to being incubated in blocking buffer solution (3% BSA, 3% normal goat serum, 0.3% Triton X100 in PBS) for 1 h. Sections were incubated overnight at 4°C with a chicken anti-GFP primary antibody (1:4000; ab13970, Abcam) in blocking buffer. The next day, sections were rinsed 3 times for 5 min in PBS and incubated for 1 h at room temperature in secondary antibody solution containing goat anti-chicken Alexa Fluor 594 secondary antibody (1:500; ab150176, Abcam) with Hoechst dye (2 µg/mL, Fisher). Afterward, sections were rinsed 3 additional times in PBS for 5 min, mounted with aqueous mounting medium, and dried overnight at 4° C. Images were acquired using an Invitrogen EVOS FL Auto Cell Imaging System.

RNAscope: Mice were anesthetized in 2-3% isoflurane and euthanized by decapitation. Brains were rapidly removed, flash-frozen in 2-methylbutane, and stored at -80°C. Brains were subsequently sectioned coronally (20 µm) on a cryostat, collected on SuperFrost Plus slides (Fisher), and stored in a slide box that was pretreated with RNAse Zap (Fisher). Before the assay, slices were fixed for 1 h at 4°C in pre-chilled 4% PFA prepared in RNase-free PBS and then rinsed twice in RNase-free PBS. Sections were then dehydrated sequentially in 50%, 70%, 100%, and 100% ethanol. Probes mixtures were prepared according to the number of targets to be labeled using a 1x diluted probe mixture for Channel 1 as diluent as follows: M1 was labeled on Channel 1

bioRxiv preprint doi: https://doi.org/10.64898/2026.10.26.761976; this version posted October 27, 2026. The copyright holder for this preprint (which was not certified by peer review) is the author/funder, who has granted bioRxiv a license to display the preprint in perpetuity. It is made available under a CC-BY 4.0 International license.

(Mm-Chrm1-O1, 98%, ACD 483441), M4 on Channel 2 (Mm-Chrm4-C2, 2%, ACD 410581-C2), and ChAT on Channel 3 (, Mm-Chat-C3) were conjugated to , 2%, ACD 408731-C3), Fluorophores used are Channel 1: TSA Vivid 570 (yellow, 1:4000), Channel 2: TSA Vivid 650 (red, 1:5000), and Channel 3: TSA Vivid 520 (green, 1:3000). The assay was performed according to the manufacturer's instructions. Following multiplex assay, sections were incubated in DAPI (RNAscope Multiplex V2 DAPI) for 30 s, and then mounted with ProLong Gold Antifade Mountant (Fisher) and cover-slipped. Sections are allowed to dry for at least 30 minutes and stored in the dark at 4°. Slides were imaged on the Leica Stellaris 5 LIAchroic Confocal System within 1 week of the assay.

RNAscope image analysis: To measure ratio of M1- and M4-positive cells to total cells, M4, M1, and DAPI channels were converted to Luminance, then to RGB respectively in Fiji (Image J), and then analyzed in QuPath. QuPath's Cell Detection was run on a non-deleted area (nucleus accumbens) with default values except for the following changes: Background Radius: 10 μm; Median Filter Radius: 2 μm; Sigma: 2 μm; Threshold: 1. Custom code was used to optimize M1 and M4 puncta detection thresholds using the non-deleted image (https://doi.org/10.5281/zenodo.23301431), and these thresholds were utilized for puncta detection in the deleted area (dorsal striatum). Afterward the area of deletion was determined utilizing Fiji's BigWarp plugin to fit fluorescent images to the corresponding atlas image and outlining the deletion.

bioRxiv preprint doi: https://doi.org/10.64898/2026.10.26.761976; this version posted October 27, 2026. The copyright holder for this preprint (which was not certified by peer review) is the author/funder, who has granted bioRxiv a license to display the preprint in perpetuity. It is made available under a CC-BY 4.0 International license.

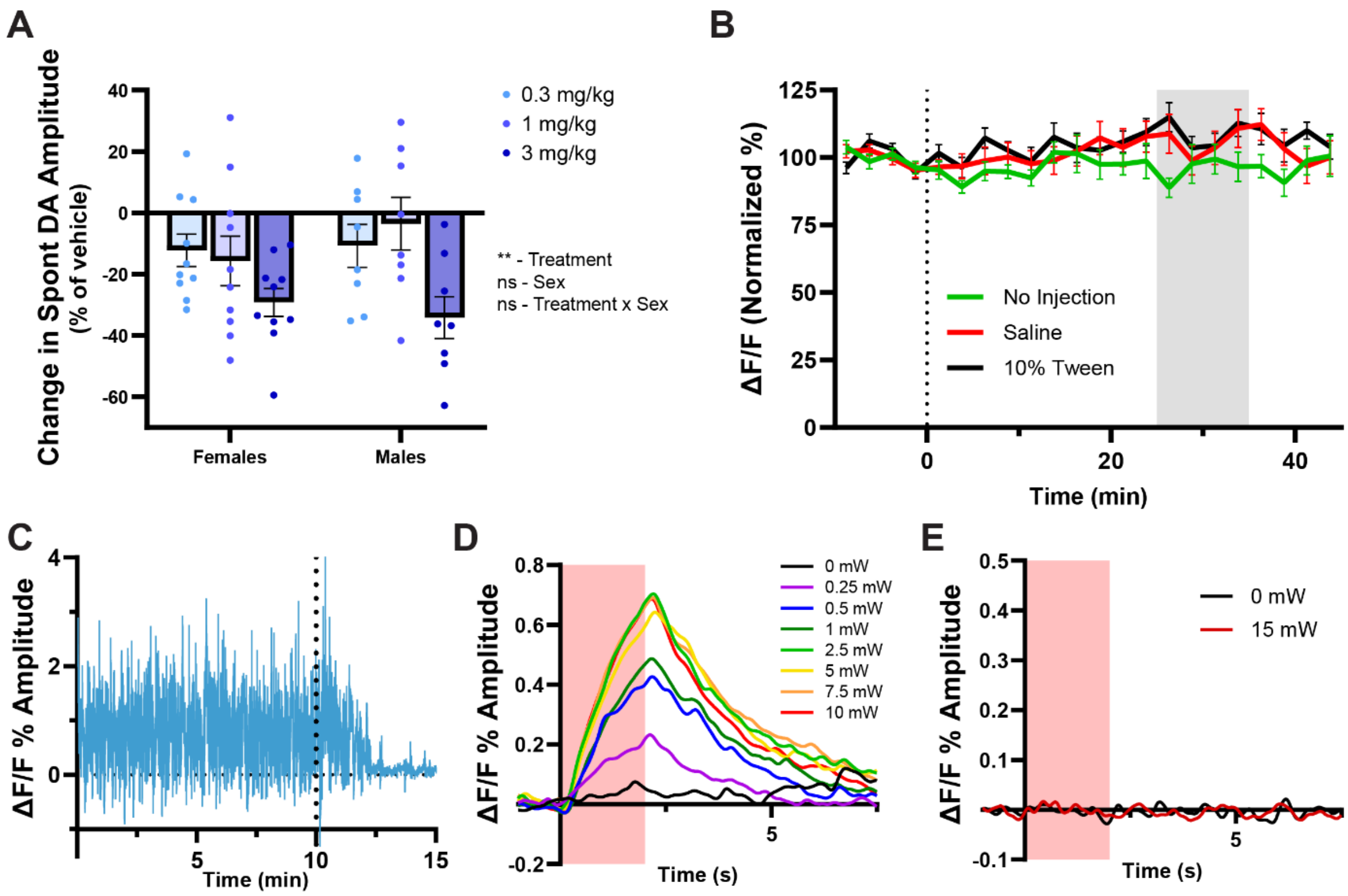

Supplemental Figure 1, related to Figure 1. (**A**) Averaged data depicting change in amplitude comparing sexes with only a main effect of treatment (two-way ANOVA, treatment: $p < 0.005$). (**B**) Time course plotting event amplitudes vehicle (n = 17) to saline (n = 16) and no injection (n = 13) depicting apparent increased amplitude following injections. (**C**) Representative trial depicting s.c. administration of 10 mg/kg D1-like antagonist SCH-23390 (dotted line), confirming signal as dLight. (**D**) Input-output curve in DAT-Cre$^{+/-}$ mice expressing dLight along with Chrimson (red portion = 20 Hz pulse duration). (**E**) Time course of optogenetic pulse applied in mice expressing dLight without Chrimson.

bioRxiv preprint doi: https://doi.org/10.64898/2026.10.26.761976; this version posted October 27, 2026. The copyright holder for this preprint (which was not certified by peer review) is the author/funder, who has granted bioRxiv a license to display the preprint in perpetuity. It is made available under a CC-BY 4.0 International license.

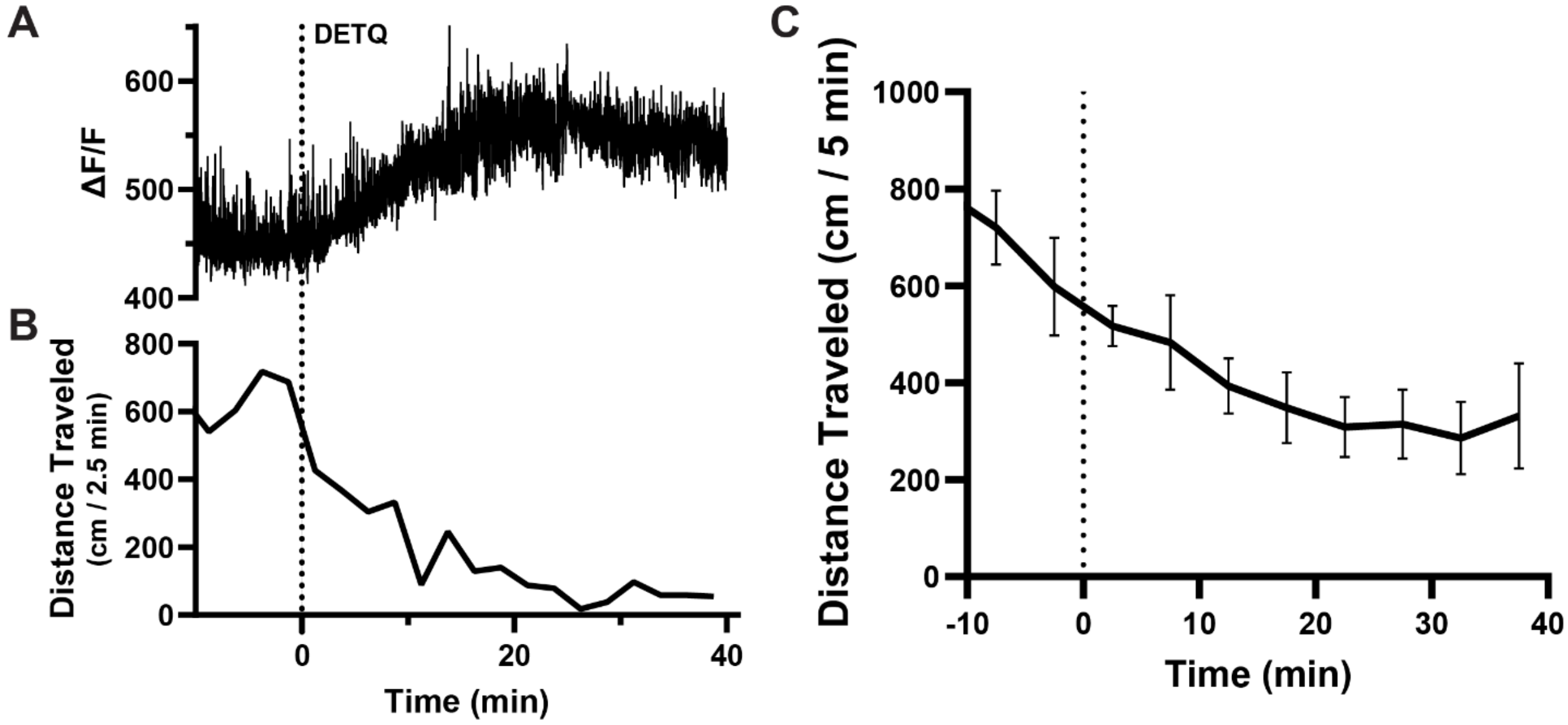

Supplemental Figure 2, related to Figure 2. (**A**) Representative trial depicting change in dLight fluorescence before and following DETQ administration (10 mg/kg; i.p.). (**B**) Representative trial depicting locomotion change over time, before and after DETQ. (**C**) Time course of distance traveled prior to and after DETQ administration with vehicle (10% Tween 80; i.p.).

bioRxiv preprint doi: https://doi.org/10.64898/2026.10.26.761976; this version posted October 27, 2026. The copyright holder for this preprint (which was not certified by peer review) is the author/funder, who has granted bioRxiv a license to display the preprint in perpetuity. It is made available under a CC-BY 4.0 International license.

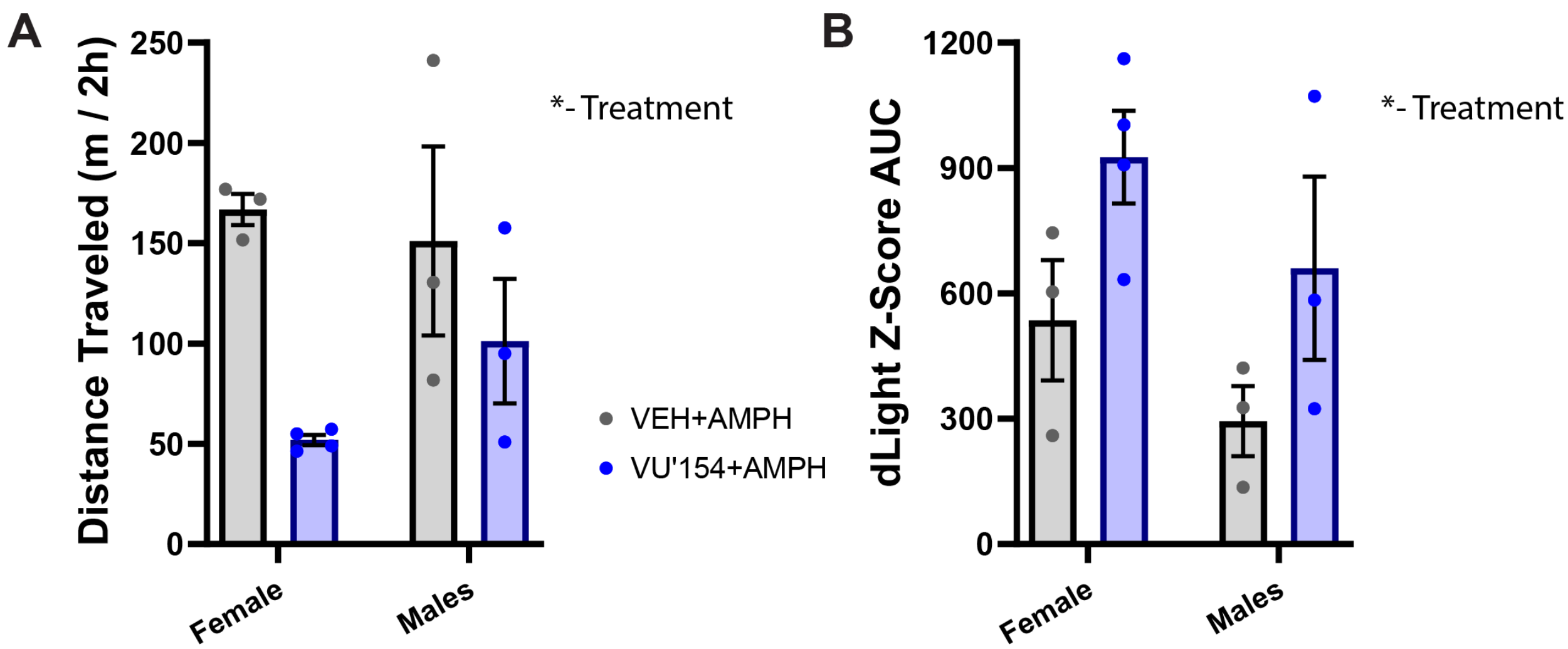

Supplemental Figure 3, related to Figure 3. (**A**) Averaged data depicting distance traveled post-amphetamine split by sex, which exhibited a main effect of treatment (p = 0.012), but no effect of sex (p = 0.535). (**B**) Averaged data depicting dLight Z-score AUC post-amphetamine, which showed a main effect of treatment (p = 0.028), but no effect of sex (p = 0.113)

bioRxiv preprint doi: https://doi.org/10.64898/2026.10.26.761976; this version posted October 27, 2026. The copyright holder for this preprint (which was not certified by peer review) is the author/funder, who has granted bioRxiv a license to display the preprint in perpetuity. It is made available under a CC-BY 4.0 International license.

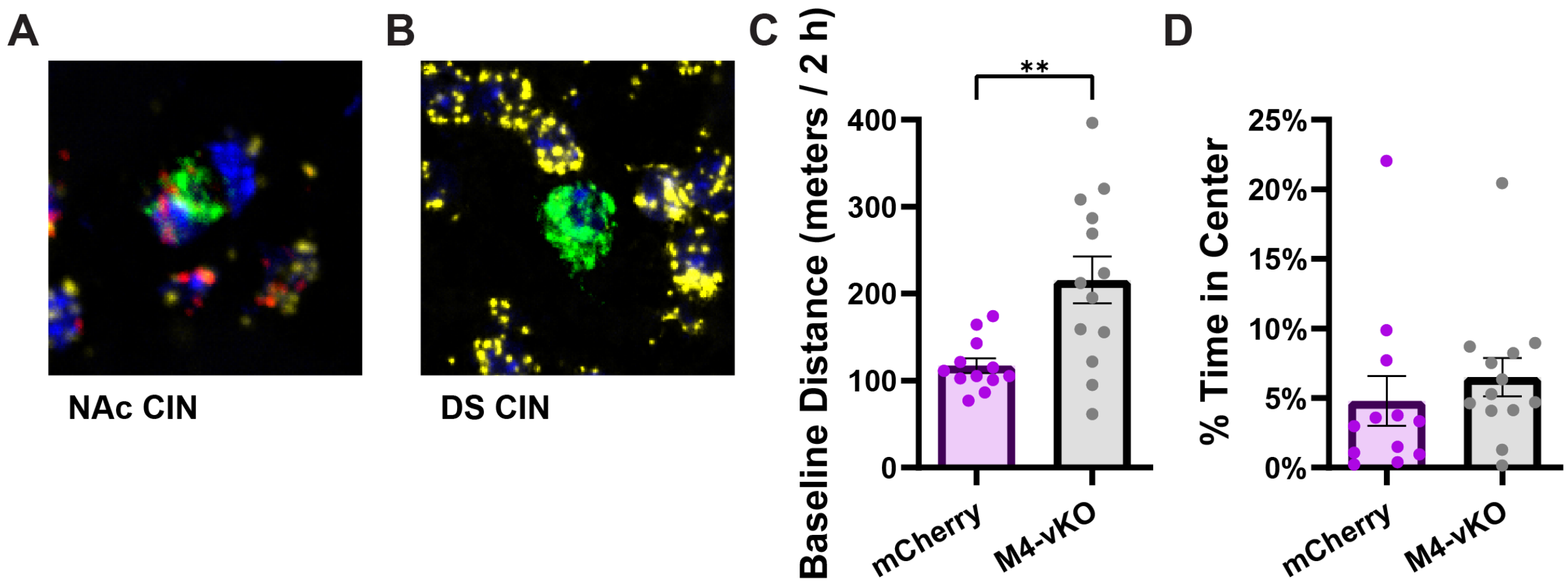

Supplemental Figure 4, related to Figure 4. (**A**) Representative image of cholinergic interneuron in the NAc expressing M4 (red). (**B**) CIN in the DS with no M4 present. (**C**) Averaged data (mean ± SEM) comparing distance traveled prior to drug administration (unpaired t-test, $p < 0.005$). (**D**) Averaged data comparing time spent in center prior to drug administration (unpaired t-test, $p > 0.05$).

bioRxiv preprint doi: https://doi.org/10.64898/2026.10.26.761976; this version posted October 27, 2026. The copyright holder for this preprint (which was not certified by peer review) is the author/funder, who has granted bioRxiv a license to display the preprint in perpetuity. It is made available under a CC-BY 4.0 International license.

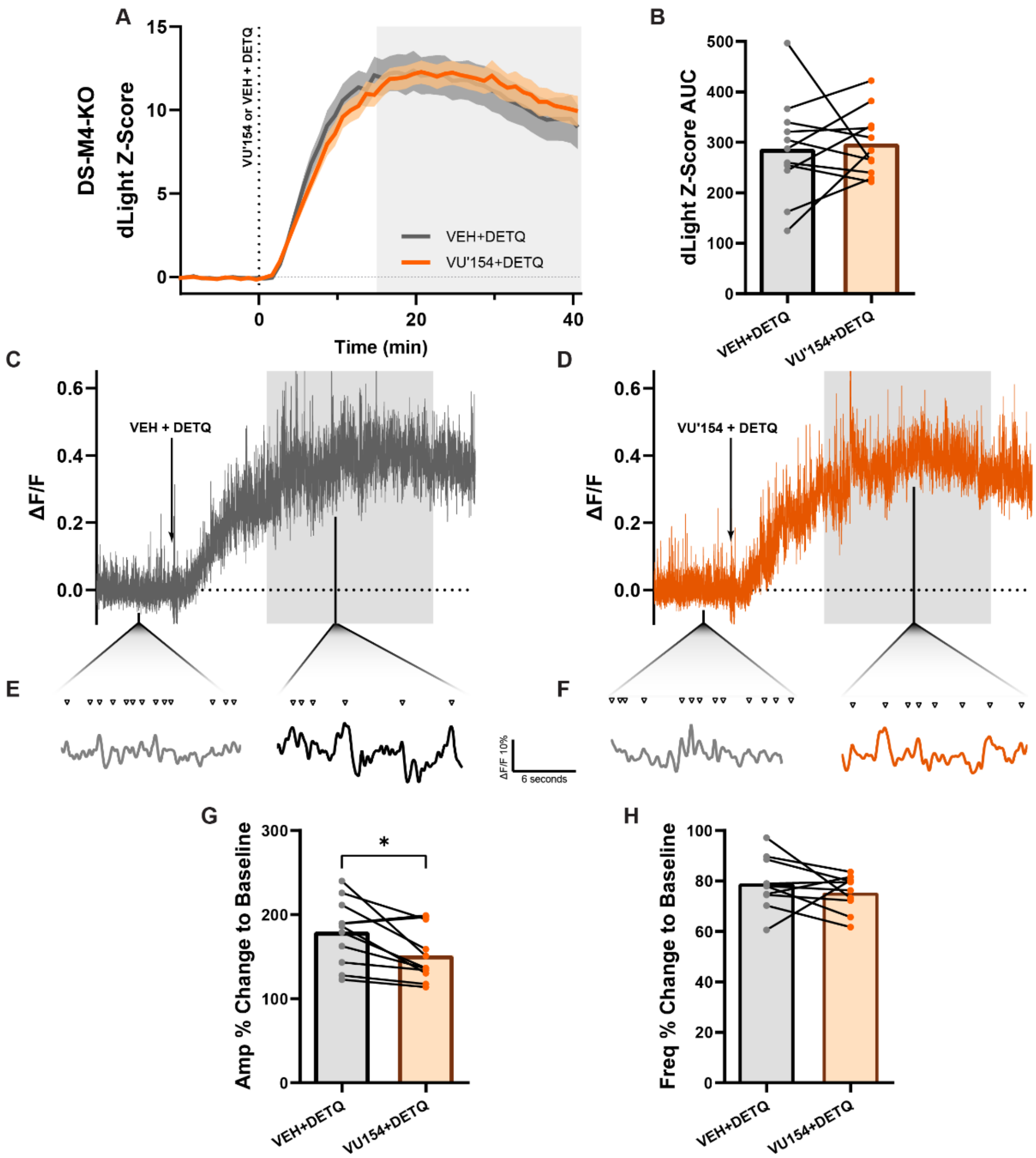

Supplemental Figure 5. (**A**) Time course of change in measured dLight fluorescence intensity (Z-score relative 10 min pre-injection. (**B**) Averaged data depicting dLight Z-score AUC (paired t-test, $p > 0.05$). (**C**) Representative trial trace following coadministration of DETQ (10 mg/kg i.p.) with vehicle and (**D**) 3 mg/kg of VU'154. (**E**) Snippets of trace labeled with detected events and base line and peak period after DETQ with vehicle and (**F**) 3 mg/kg of VU'154. (**G**) Averaged data depicting mean event amplitudes at peak period relative to baseline (within subjects) ($n = 10$, paired t-test, $p < 0.05$). (**H**) Averaged data depicting mean event frequency at peak period (paired t-test, $p > 0.05$).
